# From disorder to icosahedral symmetry: How conformation-switching subunits enable RNA virus assembly

**DOI:** 10.1126/sciadv.ady7241

**Published:** 2025-09-24

**Authors:** Siyu Li, Guillaume Tresset, Roya Zandi

**Affiliations:** ^1^ Department of Physics and Astronomy, University of California, Riverside, Riverside, CA 92521, USA.; ^2^ Université Paris-Saclay, CNRS, Laboratoire de Physique des Solides, 91405 Orsay, France.

## Abstract

Icosahedral capsids are ubiquitous among spherical viruses, yet their assembly pathways and governing interactions remain elusive. We present a molecular dynamics model that incorporates essential physical and biological features, including protein diffusion, genome flexibility, and a conformational switch that mimics allostery and activates the elastic properties of proteins upon binding. This switch makes the simulations computationally feasible, overcoming long-standing limitations of previous models. Using this framework, we successfully reproduce the self-assembly of subunits into icosahedral shells with *T* numbers greater than one—most notably *T* = 3, the most common structure in nature—a feat rigid-body models have so far failed to achieve. We also examine how genome architecture influences assembly and observe trends consistent with experiments using cowpea chlorotic mottle virus proteins: RNAs with more complex structure yield more complete capsids than do linear ones. These results establish a predictive framework for genome-guided assembly and offer insight into designing synthetic capsids for biomedical applications.

## Teaser

Conformation-switching subunits enable simulations where disordered RNA-protein complexes self-organize into symmetric shells.

## INTRODUCTION

Single-stranded RNA (ssRNA) viruses, which affect humans, animals, and plants, constitute the largest and most widespread genetic class of viruses (*1*–*7*). During their replication process, hundreds or even thousands of proteins come together to construct the protective viral shell (capsid), enclosing the genetic materials (*8*–*10*). Despite their profound impact on our daily lives, as exemplified by the recent COVID-19 pandemic, our understanding of the virus formation, both in vitro and in vivo, remains unusually limited.

Experimentally, characterizing assembly pathways through either the techniques that monitor individual capsids or bulk approaches is challenging because of the small size of the virus and the transient, short-lived nature of its intermediate structures (*11*–*15*). Because of the lack of experimental resolutions and computationally very expensive atomistic simulations for the entire capsid formation (*16*–*18*), coarse-grained computational models have been used to explore the role of various factors critical to the assembly process (*19*–*27*).

Molecular dynamics (MD) simulations using rigid triangular subunits have notably advanced our understanding of how the smallest icosahedral viral capsid, characterized by triangulation number T=1 , assembles around a genome. These models, developed by several research groups, have provided valuable insights into the principles of subunit association, nucleation, and growth, particularly for systems where geometric constraints are minimal (*28*–*35*). Note that the total number of proteins in an icosahedral structure is equal to 60T , where T is the triangulation number assuming certain integers (1,3,4,7…) (*36*). Because a T=1 structure requires only 20 triangles to form a closed icosahedral shell, its assembly pathway is relatively straightforward. In contrast, the assembly mechanisms and intermediate structures leading to T=3 and T=4 capsids—the most prevalent structures in nature—remain largely unexplored.

To our knowledge, rigid-body models have failed to date to achieve the spontaneous assembly of capsid proteins around long viral genomes into highly symmetric T=3 structures while also resolving the intermediate stages of assembly. Previous studies of T=3 capsid assembly have either focused on empty shells or used simulations with spherical cargoes, which artificially simplify the assembly pathway by imposing the capsid’s curvature (*20*, *27*, *37*). However, the mechanisms underlying the assembly and packaging of RNA or a flexible genome remain largely unexplored. It is remarkable that so many subunits can be absorbed onto a flexible genome, initially forming a disordered, irregular complex, and ultimately assembling into a stable, closed shell with icosahedral symmetry. The absence of MD simulations of T=3 or T=4 viruses in the literature highlights how the additional degrees of freedom and complexity introduced by a flexible genome make it challenging to explore the interactions and mechanisms that enable capsid proteins to overcome barriers between disordered and ordered states.

Here, we introduce a subunit design that incorporates protein flexibility through a conformational switching mechanism that mimics allosteric regulation. This enables quasi-equivalent positioning and provides a general computationally efficient framework for simulating capsid assembly via MD. The model accounts for protein diffusion and captures the packaging of a flexible genome by identical capsid subunits, leading to the spontaneous formation of T=3 structures. While our primary focus is on T=3 structures, the model also successfully assembles T=4 capsids—representing the first MD simulations to demonstrate the formation of a complete T=4 shell around a flexible genome. By including only the most essential physical and biological interactions, this allostery-inspired design makes it feasible to explore full assembly kinetics and intermediate states that were previously computationally prohibitive—significantly reducing simulation cost while preserving biologically relevant behavior.

The model captures the emergence of different triangulation numbers without relying on complex, location-specific assembly rules—such as varying subunit geometries, tuning interaction strengths, or engineering proteins to adopt distinct conformations based on their positions within the shell. Instead, it employs a single type of subunit, as is the case for most T=3 and T=4 viruses in nature, and relies solely on universal physical principles. This is consistent with observations that similar capsid symmetries arise in viruses with highly divergent amino acid sequences.

Notably, our findings reveal a virus assembly pathway involving multiple intermediate states, in contrast to previous studies that identified a narrow pathway for T=3 and T=4 viruses (*15*). We find that the most efficient pathway involves the assembly of capsid fragments at a few locations along the genome, followed by the attraction of these fragments leading to genome condensation, which facilitates subunit rearrangement (see movie S1). Counterintuitively, we discover multiple pathways in which numerous fragments—containing varying numbers of subunits and defects—merge to form a perfectly closed icosahedral shell. We note that most viruses with triangulation number T=7 or larger rely on scaffolding proteins for proper assembly. Although incorporating such features introduces additional complexity, the method presented here is sufficiently robust and flexible to address these systems; however, their exploration will be the subject of a separate study.

To illustrate the model’s capabilities, we compare the advantages of RNA packaging with those of a linear polymer using a combination of computational simulations and experimental techniques. Using small-angle x-ray scattering (SAXS) and cryo–transmission electron microscopy (cryo-TEM), we observe the assembly of T=3 structures using capsid proteins and viral RNA derived from a plant virus, as well as a nonviral RNA with a distinct topology. Our simulations capture trends that align with key features of the experimental data. The model’s robustness enables us to use MD simulations to explore and interpret a broad range of previously inaccessible experimental phenomena, including virus disassembly and genome release, packaging signals, the influence of RNA size and secondary structure, the role of protein N-terminal domains, and the geometric diversity of building blocks and heterogeneous protein subunits, to name a few.

## RESULTS

### Design principles

**Fig. 1.**
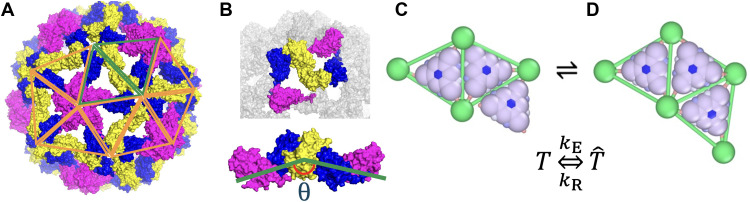
Schematic representation of subunits inspired by the capsid structure of CCMV. (**A**) A swollen cowpea chlorotic mottle virus (CCMV) capsid (PDB: 8CPY) at pH 7.5 (*62*) is displayed, with several triangles labeled to denote the subunits used in the simulations. The protein colors are assigned based on their symmetry group. (**B**) Display of two trimeric subunits, highlighting the torsional angle ( θ ) associated with protein spontaneous curvature. Recent experiments demonstrate that CCMV capsid proteins first assemble into dimers, with the subsequent addition of dimers resulting in the formation of trimers, pentamers, and hexamers. On the basis of this observation, we selected trimers as building blocks, primarily due to their ability to easily incorporate the proteins’ elasticity and spontaneous curvature, both of which are crucial for the formation of closed shells. (**C**) Two elastic subunits, denoted by $\hat{T}$ , are positioned adjacent to a rigid subunit, denoted by T . (**D**) Upon interacting with another subunit, a conformational change is triggered—mimicking an allosteric effect—whereby the subunit transitions into a bonding-capable state. This allosteric-like transition activates specific elastic interactions between subunits, analogous to how conformational shifts in proteins can enable or regulate binding in biological systems. At this stage, elasticity becomes relevant, and the subunit is enclosed by a green elastic triangle. The parameters $k_{E}$ and $k_{R}$ correspond to the effective association and dissociation rates of the subunits, respectively. Images are made using PyMOL and OVITO (*63*, *64*).

As in most previous simulations of virus assembly, we use triangular subunits as the basic building blocks (see Fig. 1) (*8*, *38*). Mohajerani *et al.* referred to triangular units as “trimers-of-dimers” and successfully used them to model the assembly of empty hepatitis B virus capsids (*38*). Triangular subunits have also been widely used in other studies to represent coarse-grained assembly intermediates (*26*, *29*, *31*, *32*, *34*). In our model, these subunits can be interpreted either as simple trimers or, following Mohajerani *et al.*, as trimers-of-dimers; however, this distinction has no impact on the final assembly outcomes. Although most viruses initially form dimers, which subsequently assemble into trimers and then into pentamers and hexamers, at this coarse-grained level, simulating the assembly of 90 dimers or 60 trimers to form a T=3 structure does not fundamentally alter the assembly pathway. In both cases, the formation process proceeds through disordered intermediate structures, and the interactions between subunits and the genome must guide the system through energy barriers to ultimately produce a stable icosahedral shell. This modeling approach captures the essential features of viral assembly while significantly reducing computational complexity. We note that while simulating the assembly of 90 dimers or 60 trimers to form a *T* = 3 structure captures similar overall geometry and intermediate states (i.e., pentamers and hexamers), the choice of different fundamental units—dimers versus trimers—can generate distinct interaction networks that may influence disassembly and the local assembly pathway (*39*).

In general, when a protein diffuses freely in solution, its elastic properties do not significantly affect the assembly process. To make our simulations computationally efficient, we therefore model free subunits as rigid bodies during their diffusion in solution. However, once a protein interacts with or attaches to another, elasticity becomes essential. This behavior is consistent with an allosteric mechanism, where interaction triggers a conformational switch that activates new bonding capabilities. The principle of quasi-equivalence—which states that identical proteins can adopt different conformations depending on their location within a capsid—further suggests that conformational flexibility, governed by such allosteric-like transitions, is crucial for a protein to occupy quasi-equivalent positions within the shell.

Accordingly, during simulations, when two free trimers diffuse into close proximity and begin interacting, we introduce elasticity by enclosing each free trimer within an elastic triangular frame (the green triangle shown in Fig. 1C). We refer to these conformationally flexible units as “elastic trimers” ( $\hat{T}$ ) and the free subunits as “rigid trimers” ( T ). This transition enables subunits to undergo a conformational change reminiscent of an allosteric response, activating specific binding capabilities upon interaction, analogous to conformational changes observed in proteins during the assembly process. Throughout this work, we use the term “allostery” to refer to conformational changes in a trimeric subunit triggered by physical interaction with another trimer, consistent with the classical definition in which structural transitions are induced by binding to a different molecule.

As two elastic trimers interact, they merge at their interfaces, and elastic bonds form between them. The energy of a growing shell includes both stretching and bending components,

$$\begin{array}{c} \begin{array}{c} \;E_{\text{elasticity}}=E_{s}+E_{b}+E_{s}^{\text{inner}}\\ =\frac{1}{2}k_{s}\sum_{i}{\left(l_{i}-l_{0}\right)}^{2}+k_{b}\sum_{i}[1-\text{cos}\left(\theta_{i}-\theta_{0}\right)\text{]}\\ \;+\frac{1}{2}k_{s}\sum_{{\hat{T}}_{i}}\sum_{j=1}^{3}{\left(l_{{\hat{T}}_{i},j}-l_{\hat{T}0}\right)}^{2} \end{array}\; \end{array}$$ (1)

where $l_{0}=3a$ is the equilibrium size of each trimer side with a=3nm the fundamental length of the system, $\theta_{0}$ is the preferred dihedral angle closely related to the spontaneous radius of curvature (*21*), $l_{i}$ and $\theta_{i}$ are the length and the dihedral angle of the bond i , and $k_{s}=100k_{B}T/a^{2}$ and $k_{b}=500k_{B}T$ are the stretching and bending moduli, respectively. Moreover, each elastic vertex, $E_{V}$ (green ball), is connected to a vertex of a rigid triangle through a ligand ( $T_{V}$ ) with equilibrium length $l_{\hat{T}0}$ , which has an elastic energy $E_{s}^{\text{inner}}$ (see Materials and Methods for more details).

Figure 1C illustrates a rigid trimer positioned near two elastic trimers. Upon interaction, the originally rigid triangle undergoes a conformational change with a certain probability, transitioning into an elastic state. This conformational switching mimics an allosteric response, where binding activates new interaction capabilities. The probability reflects the rate at which two protein subunits associate (see below). The transition results in the formation of an elastic bond between the two trimers (see Fig. 1D).

The transformation between rigid and elastic states is reversible: Trimers can dissociate and revert to rigid units. The rates of conformational change between a rigid trimer and an elastic trimer are governed by $k_{E}$ and $k_{R}$ , which correspond to the probabilities of transitioning from a rigid state to an elastic state ( $p_{E}$ ) and from an elastic state back to a rigid state ( $p_{R}$ ), respectively (see Materials and Methods for details). These rates and probabilities reflect the reaction times required for two proteins in close proximity to undergo conformational changes and interact or, alternatively, to dissociate and return to their original rigid form. With these subunits, we now have the capacity to explore stages of virus assembly that were previously inaccessible. We note that reaction rates in our simulations could, in principle, be compared with experimentally derived values using approaches similar to those used by Tresset *et al.* (*40*). However, a direct mapping would require additional parameterization, such as atomistic simulations to extract absolute timescales for protein-protein and protein-RNA interactions, as well as a systematic comparison with broader experimental datasets across different viruses and conditions, which are currently unavailable.

### Fragments of capsids join to form a perfect icosahedral shell

Using the subunits depicted in Fig. 1, we simulate protein dynamics using a Langevin integrator. The energy of the system can be written as

$$\Delta G=E_{\text{elasticity}}+E_{p-p}+E_{c}+E_{\text{ele}}-N_{T}\mu$$ (2)

where the first term is provided in Eq. 1, the second term corresponds to the protein-protein interaction between two small ligands positioned at the edges of the trimers (for particles $T_{a}$ and $T_{b}$ see Materials and Methods), and the third term denotes the interaction arising from protein conformational changes induced by allosteric effects $E_{c}=k_{B}TN_{L}\text{log}p_{R}/p_{E}$ , with $N_{L}$ the number of elastic bonds between two elastic trimers in a growing shell. The fourth term represents the electrostatic interaction between the proteins and genome, and the last term corresponds to the chemical potential, $\mu =k_{B}T\text{log}\left(c/c_{0}\right)$ , of the free proteins in solution, with c their concentration and $c_{0}$ a reference state.

**Fig. 2.**
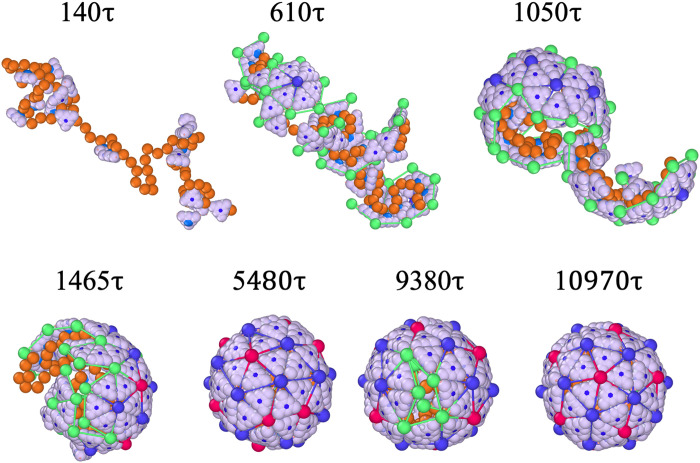
Allosteric subunits drive symmetric capsid assembly. Conformational switchable subunits activates elasticity upon binding to genome and self-organize into higher symmetric triangulation shells. The snapshots illustrate the full assembly kinetics of a T=3 particle at the protein concentration $C_{p}=100\;\mu M$ with a chain length 80a . Note that free proteins in solution are not shown to focus on the assembly process (see movie S1 for simulations including background proteins). Right at the beginning, the proteins aggregate around the genome due to the electrostatic interaction between positively charged proteins and the negatively charged linear chain. The proteins also attract each other with a strength of $\epsilon_{\text{pp}}=5k_{B}T$ . As more proteins aggregate, additional subunits transform into elastic ones capable of forming bonds, with probabilities $p_{E}=1.0$ and $p_{R}=0.1$ , corresponding to the rates of protein-protein association and dissociation ( $k_{E}$ and $k_{R}$ , respectively). The other parameters in the system are stretching modulus $k_{s}=100k_{B}T/a^{2}$ and $k_{b}=500k_{B}T$.

Figure 2 illustrates the snapshots of the formation of a T=3 structure around a linear chain with a length of l=80a at various times t with a protein concentration of $C_{p}=100\;\mu M$ . The probabilities at which the proteins undergo conformational changes are $p_{E}=1.0$ and $p_{R}=0.1$ , which controls the reversibility of the process and is related to the strength of interaction due to the protein conformational changes (see Materials and Methods for details). As noted above, these probabilities are related to the reaction times for two proteins in close proximity to undergo conformational changes and interact or, alternatively, to dissociate and revert to their free-state forms.

Throughout the simulations, an elastic trimer in an unfavorable position may transition to a rigid state with probability $p_{R}$ , allowing it to detach from the shell and diffuse back into the reservoir. Conversely, a trimer located at an energetically favorable site remains attached to the growing shell—even after transitioning to a rigid body—and may eventually revert to an elastic state. To become fully detached, a bound protein must first dissociate by converting to the rigid state, which corresponds to the removal of the elastic bond between subunits. Following dissociation, subunits can still experience hydrophobic interactions; however, the formation of an elastic bond represents a specific, conformation-dependent interaction. Thus, conformational change is required for both specific binding and detachment.

Figure 2 (see also movie S1) shows that around t=140τ , several free proteins become absorbed into the genome and aggregate without yet forming elastic bonds. We note that τ represents the system time unit, and when calibrated with our experiments, we find it to be on the order of milliseconds (*7*, *21*). As shown in the figure, around t=610τ , multiple nucleation sites emerge around the genome, forming hexamers (blue vertices and bonds) and larger oligomers. Note that the green vertices represent the elastic vertices, located only at the edge of the growing shell.

At about t=1050τ , smaller oligomers throughout the chain begin to merge, forming larger fragments that “squeeze” and encapsulate a significant portion of the genome within them. The presence of line tension, caused by the subunits having fewer neighbors at the edge, makes the intermediate states of the capsid energetically unfavorable. To this end, around t=1465τ , the fragments join and rearrange to minimize the energy associated with the edge of the growing shell. Quite interestingly, around t=5480τ , the shell is completely closed but it has an irregular shape and the “wrong” symmetry. For the remainder of the simulations, the pentamers and hexamers that initially formed in incorrect positions associate or dissociate until they eventually assemble into a perfect icosahedral shell around t=10,970τ.

**Fig. 3.**
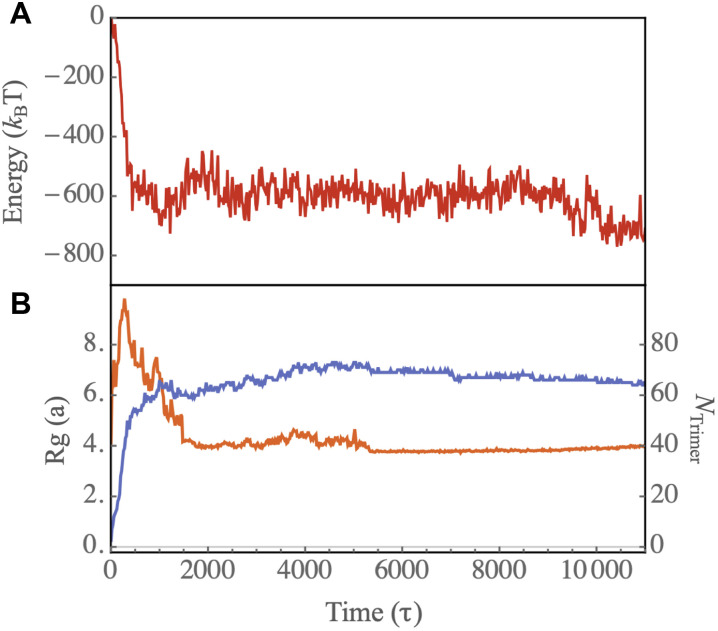
Assembly dynamics show genome condensation followed by structural ordering. The dynamics of protein-RNA assembly show rapid genome condensation followed by a gradual transition to an ordered capsid structure. (**A**) Energy of the genome-protein complex as a function of time for the assembly pathway illustrated in Fig. 2. (**B**) Number of trimers attached to the genome (blue curve) and radius of gyration of protein-RNA complex ( $R_{g}$ , orange) as a function of time. The radius of gyration includes contributions from both the genome and all proteins bound to it.

The transition from an irregular shell to an icosahedral capsid takes a rather long time as the complete shell needs to partially disassemble and many subunits have to rearrange to form a perfect shell. Figure 3A shows the total energy of a growing shell as a function of time. The figure reveals the presence of an energy barrier for this disorder to order transition, stemming from the dissociation of some subunits from the complete irregular shell (see the capsid around t=9380τ in Fig. 2).

Figure 3B shows the radius of gyration of the complex of the genome and proteins (the orange line) and the number of trimers around the genome as a function of time (the blue line). The plot reveals an initial rise in the radius of gyration, which is due to the initial configuration of the chain. As time passes, the capsid fragments join together, consequently condensing the chain. The blue plot in Fig. 3B indicates that the number of trimers quickly reaches around 60, forming an irregular closed shell. However, the transition from disorder to order within the capsid structure is a slow process, taking an order of magnitude longer to complete. Note that some T=3 capsids contain more than 60 trimers. In these cases, we observe that some subunits are enclosed within the capsid, a behavior typically associated with linear chains. In the next section, we present the results of our simulations with branched polymers, which more closely resemble the structure of RNA.

It is important to note that a key factor influencing the formation of error-free T=3 icosahedral shells is the timescale of protein binding and unbinding events, which is governed by the probabilities of conformational changes. In all simulations presented in this paper, we set the probability of transitioning from the rigid to the elastic state ( $p_{E}$ ) to 1 while varying the probability of the reverse transition ( $p_{R}$ ). This means that a bound elastic subunit can revert to the rigid state with probability $p_{R}$ , after which it may either remain attached or detach and diffuse away (see Materials and Methods for details). A protein can fully dissociate from the assembling shell only when it is in the rigid state.

**Fig. 4.**
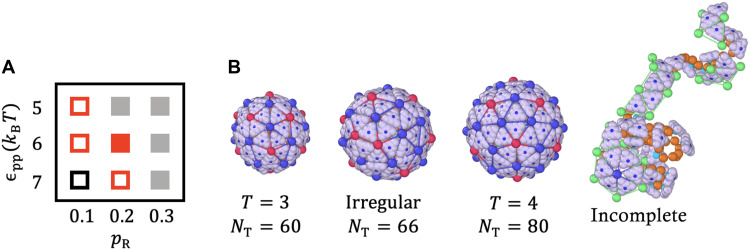
Conformational switching and protein interactions control assembly outcomes. The assembly products are governed by the probability of conformational switching and the strength of protein-protein interactions. (**A**) Shape “phase” diagram as a function of the strength of protein-protein interaction ( $\epsilon_{\text{pp}}$ ) and $p_{R}$ , which corresponds to the reaction time for protein detachment. The hollow red squares denote regions where T=3 structures were observed for less than 50% of the time, while the solid red square indicates when they were observed for more than 50% of the time. Black squares highlight areas where only T=4 or irregular shapes appear, while the gray solid region denotes instances where only incomplete shells were observed (see fig. S2 for detailed assembly morphologies). (**B**) Representative structures corresponding to the phase diagram in (A). The simulations were performed at the protein concentration $C_{p}=100\;\mu M$ , spontaneous radius of curvature $R_{0}=4.2a$ , and genome length L=80a.

The two-dimensional phase diagram in Fig. 4A illustrates how assembly products depend on the conformational switching probability $p_{R}$ and the strength of protein-protein interactions $\epsilon_{\text{pp}}$ (see Eq. 2 and Materials and Methods). Two distinct regions in phase space yield predominantly icosahedral structures: (i) small $p_{R}$ values combined with weak protein-protein interactions, and (ii) large $p_{R}$ values with strong interactions. For small $p_{R}$ (i.e., lower detachment probability), the process is less reversible, necessitating weaker hydrophobic interactions to permit error correction. A low $p_{R}$ reflects strong specific binding, likely mimicking stable post-binding conformational states (akin to allosteric regulation), which hinder dissociation. Conversely, increasing $p_{R}$ enhances the detachment rate, but sufficiently strong hydrophobic interactions can compensate, still enabling the formation of ordered capsids.

The red squares in the phase diagram of Fig. 4A indicate the regions in which we observe T=3 shells as well as some irregular structures. The difference between the hollow and filled squares is that with the filled ones, we observe a specific structure for most of the time, whereas with the empty ones, this structure is observed for less than half of the time. For instance, when $p_{R}=0.1$ and $\epsilon_{\text{pp}}=5k_{B}T$ , we observe T=3 shells, but most structures assume irregular shapes (see Fig. 4B for a representative structure). Upon further increasing $\epsilon_{\text{pp}}$ to $7k_{B}T$ , at $p_{R}=0.1$ , we observe a mix of T=4 structures and irregular shells (the black square) (see fig. S2).

For $\epsilon_{\text{pp}}=5k_{B}T$ (weak protein-protein interaction), since the number of trimers aggregated around genome is small, a high reversible rate ( $p_{R}=0.2$ ) inhibits the long-lasting formation of elastic bonds, thereby hindering capsid formation (the gray squares). However, if we increase protein-protein interactions ( $\epsilon_{\text{pp}}=6k_{B}T$ ), for $p_{R}=0.2$ , each trimer gains more chances to explore the energetically more favorable locations and we predominantly observe T=3 structures (the solid red square). An elastic trimer in an unfavorable position must first undergo a conformational change to “break” its bond, after which it can detach and diffuse back into the reservoir. In contrast, if a trimer occupies an energetically favorable position, it remains attached to the growing shell even after undergoing a conformational change, losing its elastic bond, and becoming a rigid body. Eventually, it can revert to the elastic state and continue participating in assembly. This reversibility makes the formation of perfect T=3 structures easier. A representative pathway of the formation of an icosahedral shell in this regime is shown in movie S2. At higher values of $p_{R}$ , the trimers can barely keep their elastic bonds, resulting in primarily aggregating around the genome without forming shells.

At the end of this section, we note that it is possible to monitor the assembly of multiple capsids in a solution of proteins with multiple genomes. Then, an important parameter will be the stoichiometry ratio between the concentrations of protein and genome (see fig. S7).

### Secondary structures of RNA alter the assembly products

In the previous section, we focused on the assembly of capsid proteins around a linear chain. However, due to base pairing, the structures of viral genomes deviate significantly from a linear chain. In this section, we examine the impact of RNA secondary structure on assembly outcomes and evaluate the extent to which the model reproduces experimental observations. We begin by presenting the results of our simulations, followed by highlighting the findings of our experiments.

**Fig. 5.**
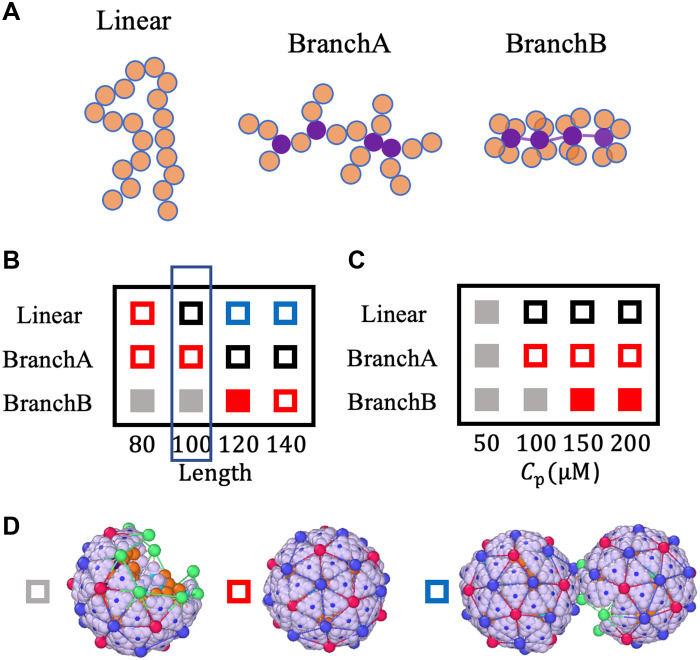
Branched RNA enhances capsid assembly efficiency. Simulations show that branched RNA structures promote more efficient capsid assembly than linear chains. (**A**) Schematic representations of RNA with various secondary structures, with branched points highlighted in purple. While in branchA-type polymer, branches are randomly distributed along the genome, in branchB type, each monomer serves as a branch point connected to four branches. Each branch consists of one monomer. (**B**) Phase diagram with different secondary structures of RNA and various genome length at a protein concentration of $C_{p}=100\;\mu M$ . (**C**) Phase diagram with different secondary structures of RNA and various protein concentrations. The genome length was set to L=100a . (**D**) Representation of an incomplete, a T=3 , and a doublet structures, corresponding to gray, red, and blue squares, respectively. The black squares in the phase diagram correspond to irregular structures; a representative shape is shown in Fig. 4B.

To investigate the impact of RNA secondary structure on assembly products, we designed three polymers with distinct topologies. Representative segments for each case are shown in Fig. 5A. The linear polymer, in the figure, contains 20 monomers without any branches. The chain denoted as branchA also comprises a total of 20 monomers, with four branching points highlighted in purple. The length of each branch is two monomers. Last, the branchB polymer also consists of a total of 20 monomers. Here, each branch has one monomer, with four branches extending from each branch point. We note that although base pairing increases the number of charges per unit length, we do not account for this effect in our model (*41*). Our focus is specifically on the geometry of the polymers. Branched polymers are more compact, leading to a higher effective volume charge density. Allowing the linear charge density to vary would introduce an additional degree of freedom that could obscure the geometric effects of branching, which we aim to isolate in this study. Because of its morphology, a highly branched polymer like branchB has a smaller radius of gyration than the less branched branchA polymer, assuming that both have the same length. It is evident that both branched polymers have smaller radii of gyration than linear polymers, indicating their greater compactness.

The impact of polymer structures of different lengths at a fixed protein concentration $C_{p}=100\;\mu M$ is depicted in the two-dimensional phase diagram presented in Fig. 5B. As before, the empty red squares represent the regime in which T=3 particles are observed before t=5000τ in at least one of the eight simulation runs (see fig. S3). For the case of the linear polymer, if the genome length is relatively short, L=80a , the icosahedral structures predominate. However, if we increase the length of genome to L=100a , we obtain irregular closed shells with no specific symmetry [Fig. 5B (black squares) and fig. S3]. Upon increasing the length further to 120a or 140a , we observe doublets (blue boxes in Fig. 5B), where two icosahedral structures, each missing one pentamer, share the chain as shown in Fig. 5D, consistent with previous experiments with different sizes of RNA molecules (*42*).

While T=3 shells cannot package a linear chain of length L=100a , they can assemble around branchA polymers of the same length. However, for longer chains, such as L=120a or 140 , capsid proteins adopt larger, irregular structures to encapsidate the branchA-type genomes (see black boxes in the phase diagram in Fig. 5B and fig. S3). Quite unexpectedly, we find that capsid subunits form incomplete shells around shorter branchB-type chains, L=80a and 100a , if the protein concentration is $C_{p}=100\;\mu M$ . This is basically due to the fact that at these lengths, the radius of gyration of the chains is comparable with the radius of a T=3 capsid. Figure 5B also shows that upon increasing the length of genome to L=120a or 140a , for the branchB-type chain, we obtain T=3 structures again.

To explore the interplay of genome structure and protein concentration for a fixed chain length of L=100a , we vary the protein concentration and observe how the structures depicted in the second column of Fig. 5B (enclosed within a narrow blue rectangle) change. The resulting phase diagram is displayed in Fig. 5C. We find that at a lower protein concentration, $C_{p}=50\;\mu M$ , the capsids remain incomplete even at t=5000τ . As before, the filled rectangles mark the regions where a particular structure was observed at least 50% of the time. Upon increasing the protein concentration, the shells enclosing linear chains remain irregular; however, we do not observe any changes for both branchA and branchB polymers. The productivity of T=3 shells increases dramatically when packaging branchB (highly branched) at high protein concentrations, as illustrated by filled red squares in the phase diagram (Fig. 5C) (see also fig. S4). It is important to note that the structures formed around branchB genomes are robust and insensitive to other parameters in the system including the probability of protein conformational changes $p_{R}$ and the spontaneous radius of curvature of proteins $R_{0}$ (see fig. S5).

**Fig. 6.**
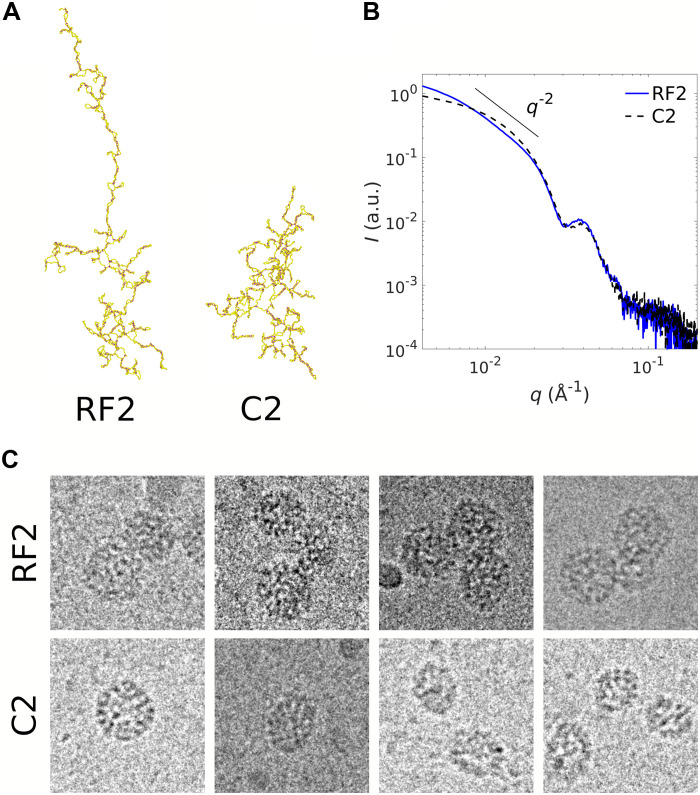
Branch-rich RNAs yield more compact viral shells. Experiments reveal that branch-rich RNA structures form more compact and efficient viral shells. (**A**) Secondary structures of RNA RF2 and C2. The structures are obtained using the ViennaRNA package (*41*, *65*) and visualized using OVITO. (**B**) Rescaled SAXS intensity of structures obtained with CCMV capsid proteins at 25 μ M in the one hand, and RF2 (blue solid line) or C2 (black dashed line) in the other hand, with a protein-to-RNA mass ratio of 4.5. Note that a mass ratio of 4.0 corresponds to 180 capsid proteins ( T=3 ) for one RNA chain. (**C**) Cryo-TEM images of structures assembled with CCMV capsid proteins at 75 μ M, and either RF2 or C2 with a protein-to-RNA mass ratio of 6.0. The leftmost image with C2 is a native-like T=3 nucleocapsid. Images are all 80 nm by 80 nm.

Several features given by our model are consistent with experimental observations. In vitro assembly experiments with cowpea chlorotic mottle virus (CCMV)—a nonenveloped, ssRNA plant virus that forms T=3 structures—are carried out by mixing purified capsid proteins with two types of RNA. RNA C2 is the second genomic RNA segment of CCMV and has 2767 nucleotides. RNA RF2 is a nonviral RNA segment with a similar length of 2687 nucleotides. Figure 6A shows the secondary structures of RF2 and C2, which were obtained using the ViennaRNA package (*43*). The figure shows that C2 has a branched conformation akin to the branchB chain of our simulations, whereas RF2 is more extended and can be assimilated to branchA or the linear chain. SAXS measurements of structures obtained with RF2 and C2 (Fig. 6B) reveal close morphologies from medium to high wave numbers ( q>0.02 Å^−1^), i.e., across length scales smaller than the size of a T=3 capsid (≃30 nm). The SAXS patterns at small wave numbers indicate that structures formed with RF2 display slight aggregation, resembling multiplets, as evidenced by a $q^{-2}$ scaling (Fig. 6B). This scaling is reminiscent of a freely jointed chain structure. Cryo-TEM images confirm the presence of a significant number of doublets and even triplets (Fig. 6C) with RF2, as predicted in our simulations for linear chains (see Fig. 5D). By contrast, we observe well-dispersed T=3 structures when C2 is used (see fig. S6), although they are coexisting with a number of incomplete capsids (Fig. 6C). This is again in line with our simulations wherein 120-long branchB chains yield most icosahedral structures (Fig. 5B). While additional experiments are needed for a quantitative comparison, it is notable that varying degrees of genome branchiness give rise to distinct capsid morphologies.

On the basis of both experiments and simulations, we find that the secondary structure of RNA influences the resulting assembly products. Specifically, there is qualitative agreement between the two: When the RNA has fewer branch points—that is, when it is less branched—the assembly yields more incomplete shells, doublets, or closed irregular shells than icosahedral shells.

## DISCUSSION

While ongoing experiments continue to shed light on viral assembly, the transient nature of intermediate states and the nanometer scale of the structures present persistent challenges for direct observation. Despite the prevalence of T=3 structures among ssRNA viruses—and decades of experimental and computational work—no published simulations have demonstrated the spontaneous formation of a complete T=3 capsid around a flexible genome. This long-standing gap has hindered our understanding of how symmetry and stability emerge during genome-guided self-assembly.

To address this, we developed a model featuring subunits that diffuse freely in solution and undergo conformational changes upon interaction, forming bonds and acquiring elastic properties. These elastic properties activate only after binding—an approach that notably reduces computational cost and enables access to full assembly kinetics previously inaccessible. Using this model, we simulate the spontaneous assembly of subunits around a flexible polymer, leading to the formation of well-ordered, stable T=3 and T=4 capsids.

Our simulations reveal important characteristics of the assembly process: Capsid proteins rapidly accumulate around the genome, driven by electrostatic interactions, often forming multiple partial capsid fragments. Although it may seem unlikely that irregular intermediates—differing in size and structure—could self-organize into a complete shell, we observe that they frequently merge into a single, closed capsid. Even under high protein concentrations, where malformed intermediates are more common, the shell often undergoes self-correction, with misassembled pentamers rearranging to restore symmetry. This transition from disorder to order unfolds over extended timescales and may require local disassembly to overcome energetic barriers. We note that the model allows for the incorporation of protein accumulation dynamics (*44*), which could potentially give rise to a nucleation-growth mechanism. For example, a time-dependent influx of trimers could be implemented via a grand-canonical approach to mimic the in vivo translation process. While not the focus of the current study, such extensions offer a promising direction for future work, particularly in models that incorporate packaging signals to explore pathway selection and other aspects of the viral life cycle.

Our findings demonstrate that conformational flexibility—introduced via an allosteric-like switching mechanism—and minimal physical interactions are sufficient to reproduce the geometric complexity of native viral capsids. The ability of capsid proteins to adopt quasi-equivalent positions and assemble into shells with different symmetries under various in vitro conditions (*22*) underscores the importance of conformational switching in mediating their interactions. The widespread ability of capsid proteins—with diverse sequences—to form icosahedral shells and package genomes under varied in vitro and in vivo conditions further suggests that virus assembly is governed by universal physical principles. These results also highlight key limitations of earlier models, which often relied on empty shells, rigid subunits, or spherical cargos (*21*, *28*, *37*), and therefore did not fully capture the assembly pathways and outcomes of T=3 capsids packaging a flexible genome.

Here, we also performed in vitro experiments to examine the assembly products of CCMV coat proteins mixed with two RNAs exhibiting distinct secondary structures. As shown in Fig. 6A, one RNA (C2) is highly branched, while the other (RF2) adopts a more linear configuration. The experimentally observed assembly products are shown in Fig. 6C, and the corresponding simulation results are presented in Fig. 5D. Both highlight the influence of RNA secondary structure on the morphology of the final capsid structures. Specifically, when the RNA has fewer branch points, the assembly tends to yield more incomplete shells, doublets, or closed irregular structures, rather than well-formed icosahedral capsids. Our simulations qualitatively reproduce these outcomes, suggesting that the model captures key aspects of how genome architecture affects assembly. This agreement supports the relevance of our coarse-grained, dynamic framework in describing genome-guided capsid formation. Further experimental work will be needed to support a detailed quantitative comparison, which will be the focus of a future study.

Moreover, our phase diagrams (Fig. 5, B and C) provide insight into how viruses may selectively package their native RNA within the crowded cytoplasmic environment, where many competing RNAs and other anionic biomolecules are present. While our model is capable of packaging linear polymers, consistent with experimental observations (*45*), the number of monomers in linear polymers must be smaller than in the structured RNA used in our study (see Fig. 5A). The increased flexibility of linear polymers can facilitate their packaging; however, longer chains—characterized by larger radii of gyration—are encapsidated less efficiently, exhibiting lower production rates and requiring longer timescales to reorganize into an icosahedral shell. We find that long, linear RNAs tend to promote the formation of incomplete or defective structures, whereas long, highly branched RNAs favor the assembly of regular icosahedral shells. This may explain the evolutionary preference for highly branched viral genomes in many RNA viruses: Enhanced compaction due to branching can lead to assembly products that are statistically less defective and energetically more stable, providing both a structural and evolutionary advantage.

Armed with this model, we are now positioned to explore several previously inaccessible questions, including virus disassembly and genome release dynamics, head-to-head competition among RNAs with distinct structures (*46*, *47*), the role of protein N-terminal domains (*23*, *32*, *48*), the influence of alternative building block geometries (*49*, *50*), the formation of larger icosahedral shells in the presence of scaffolding proteins or heterogeneous subunits, and the effect of packaging signals (*51*–*56*).

Modeling packaging signals will require introducing a form of allostery, in which protein conformational switching is triggered by localized RNA secondary structures. This type of context-dependent activation may lead to more deterministic assembly pathways and restrict the range of accessible intermediates. Moreover, in packaging signal–mediated assembly, where allostery is driven by specific RNA-protein interactions, the timescales of binding and unbinding events will remain critical determinants of assembly fidelity and efficiency.

The model can also be extended to investigate the phase behavior of empty virions as a function of pH and ionic strength (*57*), the conditions under which linear polymers are encapsidated (*45*) and alternative pathways for the encapsidation of short RNAs (*58*), with potential implications for drug delivery applications. To construct different *T*-numbers within a single solution—similar to the experiments by Comas-Garcia *et al.* (*58*) using CCMV capsid proteins and short RNA segments—we will introduce an additional allosteric conformational switch. This feature will allow subunits to adopt different conformations upon interacting with genomes of varying compactness (e.g., smaller radii of gyration) and will be a focus of future work currently underway.

Understanding how nucleic acid and capsid protein properties promote virus assembly could inspire the development of antiviral drugs aimed at disrupting viral replication, and it is also crucial for packaging peptides or other macromolecules for drug and gene delivery applications.

## MATERIALS AND METHODS

### Numerical simulations

**Fig. 7.**
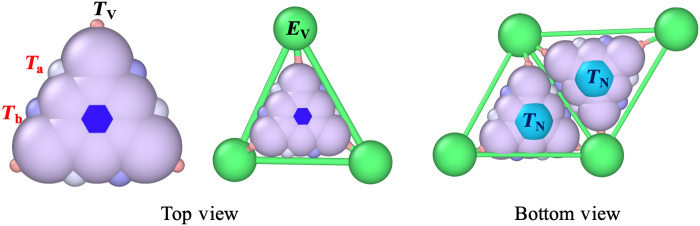
Top view of rigid and elastic trimers. Illustration of the top view of the outer surface (epitope) of a rigid trimer (left) and an elastic trimer enclosed in a green triangle (middle). The rigid trimers consist of a particle denoted $T_{M}$ , represented by a dark blue hexagon positioned at the trimer’s center of mass, along with nine constituent particles ( $T_{C}$ ). Additionally, the triangular subunits include three pairs of ligands ( $T_{a}$ , $T_{b}$ ) located along the trimers’ edges and three vertex ligands ( $T_{V}$ ). As a rigid subunit transitions to an elastic state, a triangle with three elastic sides and vertices ( $E_{V}$ ) wrap around the rigid subunit. Each elastic vertex binds to the ligand $T_{V}$ . The particle $T_{N}$ (light blue) is located on the the inner (hypotope) surface of all trimers. The $T_{N}$ particles carry nine positive charges, which interact with the negative charges on genome monomers. The radius of particles $T_{V}$ , $T_{a}$ , $T_{b}$ , $T_{C}$ , $T_{N}$ , $T_{M}$ , and $E_{V}$ are 0.1a , 0.2a , 0.2a , 0.5a , 0.5a , 0.5a , and 0.5a , respectively.

To study the virus assembly pathways, we perform MD simulations using the HOOMD-blue package (*59*). The system is initialized with N trimers in the rigid state ( T ) and one genome with a length of L. Figure 7 shows the structure of each trimer.

The hydrophobic interaction between the trimers is such that the ligand $T_{a}$ of one trimer attracts the ligand $T_{b}$ of another trimer. The interaction between ligands $T_{a}$ and $T_{b}$ can then be written as

$$\begin{array}{c} U_{\text{pp}}^{\text{LJ}}\left(r\right)=\left\{\begin{array}{cc} 0.5*\epsilon_{r}{\left(\sigma -r\right)}^{2}\;-\epsilon_{\text{pp}}, & \text{if}\;r<\sigma ;\\ \epsilon_{\text{pp}}\left[{\left(\frac{\sigma }{r}\right)}^{12}\;-2{\left(\frac{\sigma }{r}\right)}^{6}\right], & \text{if}\;r\geq \sigma \end{array}\right. \end{array}$$ (3)

where the repulsive part of the Lennard Jones (LJ) potential is replaced by a soft harmonic repulsive potential. Here, $\epsilon_{\text{pp}}$ represents the interaction strength, while $\epsilon_{r}$ stands for the repulsive strength, which we keep constant, $\epsilon_{r}=100k_{B}T$ in all simulations. $\sigma =R_{i}+R_{j}$ is the optimal distance between two particles, with $R_{i}$ and $R_{j}$ representing the radii of particles indexed i and j , respectively, and r indicating the distance between any two particles. The interactions are subject to a cutoff distance $r_{\text{cut}}=2.4a$ , with a=3nm the fundamental length of the system. All other particles in the system interact through the soft harmonic repulsive potential

$$U^{\text{rep}}\left(r\right)=0.5*\epsilon_{r}{\left(\sigma -r\right)}^{2}$$ (4)

which is subject to a cutoff distance $r_{\text{cut}}=R_{i}+R_{j}$.

The electrostatic interaction between two particles i and j is modeled by the Debye-Hückle potential

UijDH(r)=kBTZiZjlbeκ(Ri+Rj)(1+κRi)(1+κRj)e−κrr (5)

where the Bjerrum length $l_{b}=e^{2}\beta /4\pi \epsilon_{0}\epsilon$ is a measure of the dielectric constant ϵ of the solvent and is about 0.7 nm for water at room temperature. $Z_{i}$ and $Z_{j}$ represent the number of charges of particle i and j , respectively, while $\kappa^{-1}=0.5a$ is the Debye screening length. In all simulations, we considered that the number of charges on trimers located on $T_{N}$ partices is $Z_{T_{N}}=+9$ (see Fig. 7).

We model RNA as a negatively charged linear chain composed of L beads whose diameter is 1.0a and that are connected by harmonic bonds with a stretching modulus of $500k_{B}T/a^{2}$ and equilibrium length of 1.0a . We assumed that the number of charges on each bead is $Z_{C}=-9$ . The chain is the so-called Gaussian chain if we do not consider the electrostatic repulsion. We also took into account the influence of RNA’s secondary structure by modeling it as a branched polymer (see Fig. 5).

We simulate the protein dynamics through Langevin integrator, with a time step dt=0.0005τ . At every 200 steps, we check the distance between any pairs of ligands $T_{a}$ and $T_{b}$ . When the distance between two rigid trimers is less than a cutoff distance $D_{\text{cutI}}=0.45a$ , we randomly choose one of the trimers and transform it to an elastic subunit with a probability $p_{I}=0.1$ . In a different scenario where a rigid trimer moves close to an elastic trimer, we calculate the distance between the ligand $T_{v}$ of the rigid trimer and the vertex ( $E_{V}$ ) of the elastic trimer. If the distance between them is less than a cutoff distance $D_{\text{cutA}}=1.0a$ , we transfer the rigid trimer to an elastic one with a probability $P=p_{E}$ . We consider that $p_{I}<p_{E}$ , under the assumption that elastic protein subunits facilitate the transition of neighboring subunits from a rigid to an elastic state.

In situations in which an elastic trimer moves away and is not in the vicinity of any other trimers, it can switch back from elastic to a rigid one with a probability of $p_{R}$. Figure 4 shows the different values of $p_{R}$ that we have used in our simulations. There is also another mechanism for elastic trimers to become rigid subunits: We randomly choose one of the elastic trimers and transfer it back to a rigid trimer with a probability $P=p_{R}^{n}$ , where n is the number of edges that the selected elastic trimer shares with its neighbors. For example, for an elastic trimer on the edge sharing a bond with another trimer, the probability of transforming to a rigid trimer is $p_{R}$ , while if the elastic trimer is buried inside the growing shell having three neighbors, the probability will be $p_{R}^{3}$.

Another crucial step in our simulations involves the merging of subunits. If any two elastic vertices $E_{V}$ are close to each other or, more specifically, if the distance between them is less than $D_{\text{cutM}}=1.0a$ , we merge the two elastic vertices, resulting in the formation of a pentamer or hexamer, or the merging of partial shells into one piece.

For all simulations presented in this paper, we set the probability $p_{E}$ to 1. This choice is made under the assumption that each rigid subunit gains energy of approximately Δg ranging between −1 to $-\;3k_{B}T$ when transitioning from a rigid state to an elastic state and associating to an elastic subunit. Given that the Boltzmann factor associated with this change is proportional to the exponential term $e^{-\beta \Delta g}>1$ , we consistently set $p_{E}$ to 1. Correspondingly, the probability $p_{R}$ is chosen to be equal to $e^{\beta \Delta g}$ , where Δg is the energy gain associated with protein conformational changes, ranging between −1 and $-\;3k_{B}T$ . For this range, $p_{R}$ falls within [0.1,0.3] . The energy gain Δg resulting from protein conformational changes can arise from various factors. For instance, such changes may expose more hydrophobic regions, leading to an additional attraction between proteins. Alternatively, conformational changes can create the specific interactions due to morphological alterations in proteins, thereby promoting interactions between them.

We note that based on the probabilities given above, the rate constants at which the proteins undergo conformational changes can be expressed as $k_{E}=\nu p_{E}N_{T}$ and $k_{R}=\nu p_{R}^{n}N_{\hat{T}}$ (see Fig. 1). Here, $N_{T}$ and $N_{\hat{T}}$ represent the number of rigid and elastic trimers available for transition, respectively, and $\nu =10\tau^{-1}$ denotes the transition frequency, with τ being the system time unit.

It is worth mentioning that all simulations presented in the paper were conducted in a protein solution with only one genome present. To replicate in vitro experimental conditions, we placed multiple chains within the protein solution. Since monitoring the formation of several T=3 particles is computationally expensive, we focused instead on the formation of several T=1 capsids. To this end, we chose the preferred dihedral angle and the size of the genome to be commensurate with T=1 capsids. Using a protein concentration of $C_{p}=100\;\mu M$ , the stoichiometric ratio of protein to RNA is 200:8 , a genome length of l=20a , and 200 trimers, we monitored the formation of several T=1 structures, which contained 20 trimers.

As time progressed, we observed a rapid absorption of proteins onto each genome, which brought trimers closer together and initiated their transformational change into elastic structures (see movie S3 for the dynamics). Figure S7A records the number of proteins around each genome as a function of time, where we define each genome-trimer complex as a cluster. We observe that the formation pathway of each cluster is different, despite the fact that the shell comprises only 20 triangles. For instance, four clusters grew much slower, displaying a plateau around 10 to 15 trimers. The simulation snapshots at t=805τ capture two different pathways. As shown in fig. S7B, four clusters form closed capsids, whereas the other four clusters form only half shells. One important quantity in these simulations is the stoichiometry ratio of genome to protein concentrations. If we increase the protein concentrations, all clusters will eventually form T=1 structures. These simulations confirm that the kinetic pathways of multi-shell assembly closely resemble those of a single shell.

### Experimental methods

CCMV is purified from infected cowpea leaves (*Vigna unguiculata*) (*60*). Purified virions are disassembled through overnight dialysis against 0.5 M CaCl_2_, 1 mM EDTA, 1 mM dithiothreitol, 0.5 mM phenylmethylsulfonyl fluoride, and 50 mM tris-HCl (pH 7.5). Capsid proteins are then pelleted by ultracentrifugation at 150,000*g* for 18 hours and stored at 4°C in 0.5 M NaCl and 50 mM tris-HCl (pH 7.5) until use. RNA transcription is performed with the MEGAscript T7 Transcription Kit (Thermo Fisher Scientific). Freshly synthesized RNAs are purified with a MEGAclear Transcription Clean-Up Kit (Thermo Fisher Scientific) and redispersed in ultraPure deoxyribonuclease/ribonuclease-free distilled water (Invitrogen, Carlsbad, CA). Assembly is carried out by dialyzing a mixture of CCMV capsid proteins and RNA against 0.1 M NaCl and 50 mM tris-HCl (pH 7.5) overnight.

SAXS measurements are carried out at the ID02 beamline of the European Synchrotron Radiation Facilities (Grenoble, France). The sample-to-detector distance is set to 2 m, which provides q values ranging from $3.4\times 10^{-3}$ to 0.38 Å^−1^. The two-dimensional scattering images are radially averaged and further processed with the SAXSutilities package (*61*).

For cryo-TEM, 4 μ l of solution is deposited on a glow-discharged Quantifoil R2/2 grid before being plunged into liquid ethane using an FEI Vitrobot. The cryofixed samples are imaged with a JEOL JEM-2010 microscope equipped with a 200-kV field emission gun and a Gatan Ultrascan 4K charge-coupled device (CCD) camera.
