# Supplementary material for: From disorder to icosahedral symmetry: How conformation-switching subunits enable RNA virus assembly
Source: Sci Adv. 2025 Sep 24;11(39):eady7241. doi: 10.1126/sciadv.ady7241 (PMC12459405; doi:10.1126/sciadv.ady7241)
Supplement: Supplementary file 1 — Figs. S1 to S7 Legends for movies S1 to S4 [file sciadv.ady7241_sm.pdf]

Science Advances
AAAS

Supplementary Materials for

From disorder to icosahedral symmetry: How conformation-switching subunits enable RNA virus assembly

Siyu Li *et al.*

Corresponding author: Roya Zandi, roya.zandi@ucr.edu

Sci. Adv. **11**, eady7241 (2025)
DOI: 10.1126/sciadv.ady7241

The PDF file includes:

Figs. S1 to S7
Legends for movies S1 to S4

Other Supplementary Material for this manuscript includes the following:

Movies S1 to S4

Supplementary Figure

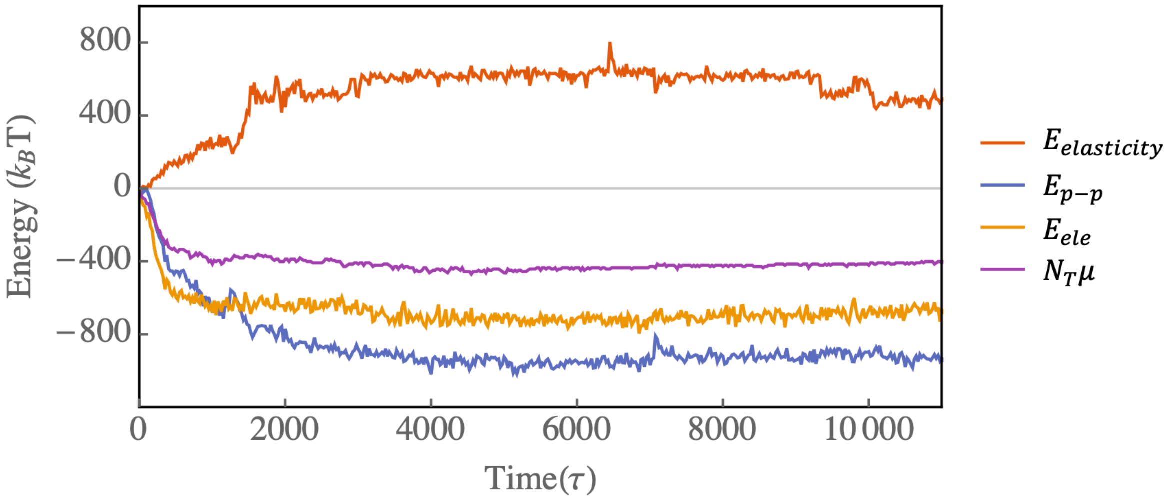

Figure S1: **Energy evolution during assembly.** Plots of various energies as a function of time for the assembly pathway shown in Fig. 2. The plots correspond to the elastic energy ($E_{elasticity}$), the protein-protein attractive interaction energy (E_{p-p}), the electrostatic interaction energy (E_{ele}), and the chemical potential ($N_T\mu$). The total energy is plotted in Fig. 3.

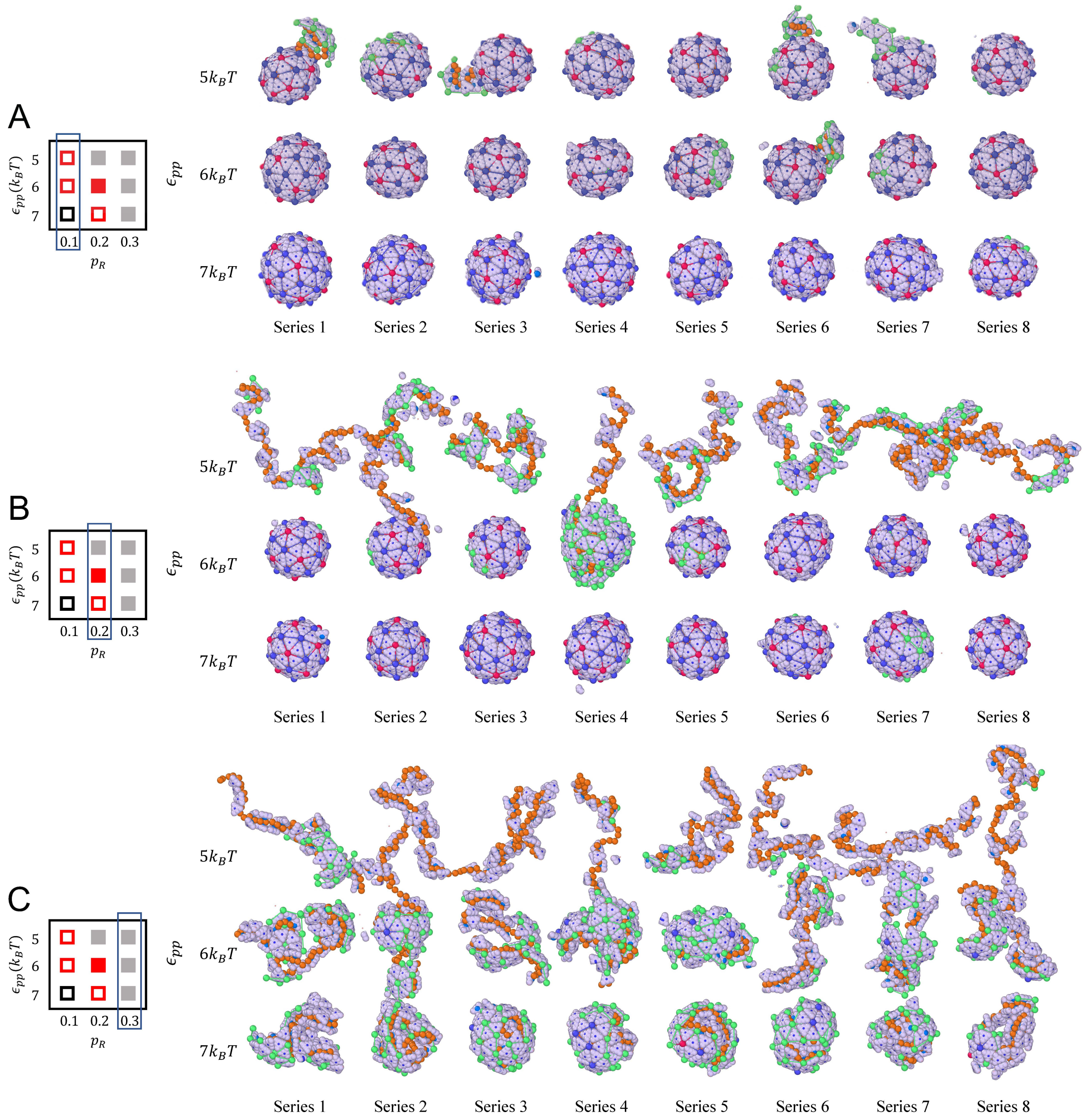

Figure S2: **Snapshots of eight simulation runs observed at** $t = 5000\tau$**.** The snapshots correspond to (A) the first column, (B) the second column, and (C) the third column of the phase diagram shown in the figure (the same as Fig. 4) with various protein-protein interactions for $p_R = 0.1, 0.2,$ and 0.3. The protein concentration used is $C_p = 100\mu M$, and chain length is $80a$. The red squares in the phase diagram indicates the regions in which we observe at least one $T = 3$ before $t = 5000\tau$.

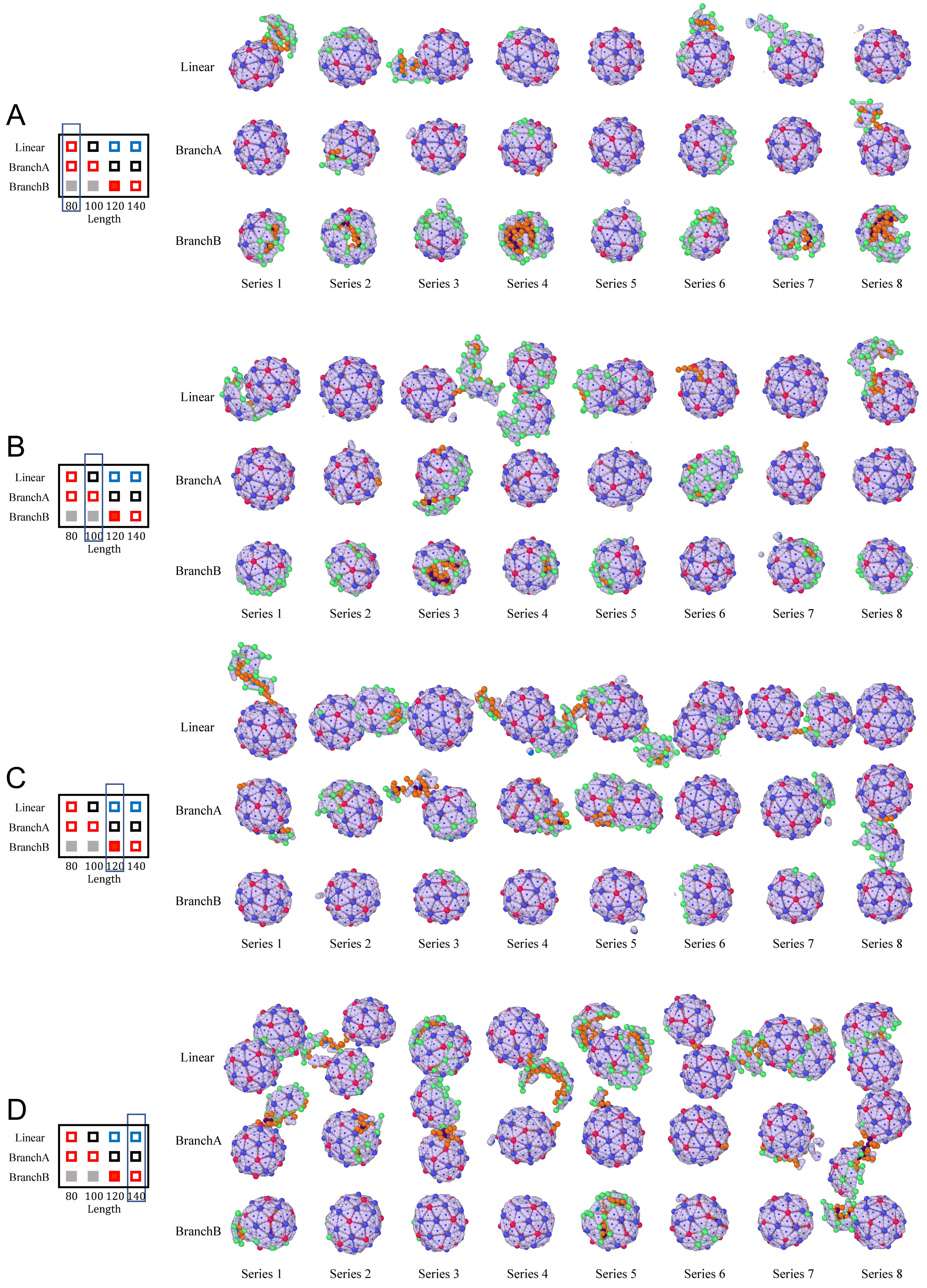

Figure S3: **Snapshots from eight independent simulation runs observed at $t = 5000\tau$.** The snapshots correspond to (A) the first column (B) the second column, (C) the third column, and (D) the forth column of the phase diagram given in Fig. 5B, with three different secondary structures of RNA. The genome lengths are $80a$, $100a$, $120a$, and $140a$. The protein concentration used is $C_p = 100\mu M$, the protein-protein interaction strength is $\epsilon_{pp} = 5k_BT$ and $p_R = 0.1$.

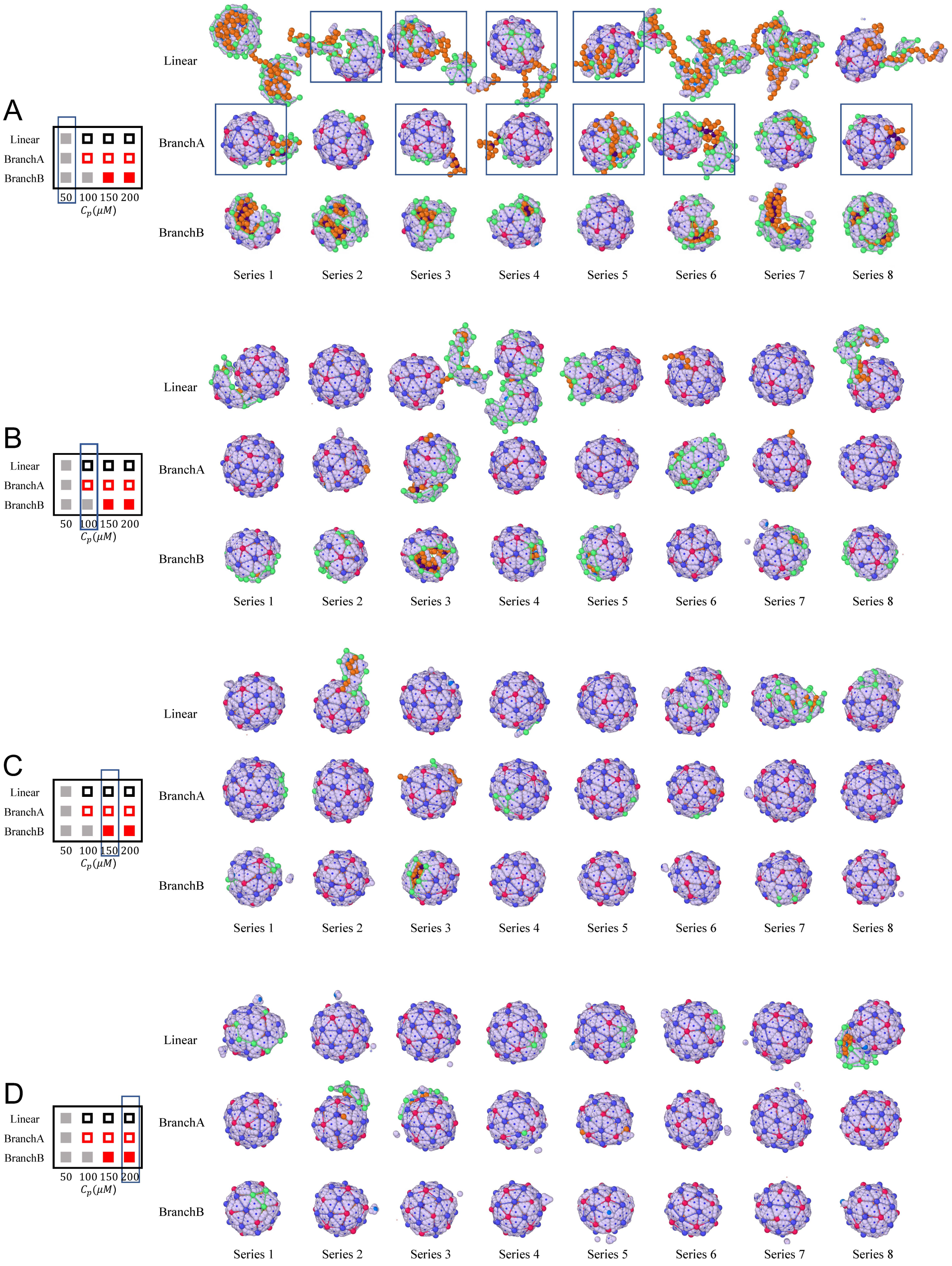

Figure S4: **Snapshots of eight simulation runs observed at** $t = 5000\tau$**.** The snapshots correspond to (A) the first column (B) the second column, (C) the third column, and (D) the forth column of phase diagram given in Fig. 5C, with three different secondary structures of RNA and various protein concentrations of $C_p = 50\mu M$, $100\mu M$, $150\mu M$, and $200\mu M$. The genome length is $L = 100a$ and the protein-protein interaction strength is $\epsilon_{pp} = 5k_BT$ and $p_R = 0.1$. Note that ten snapshots in (A) are marked by square boxes. These incomplete shells will later form closed $T = 3$ particles if we increase the simulation time. In certain cases that even if the shells are closed, the genomes are not fully packaged; they remain partially exposed outside of the shells.

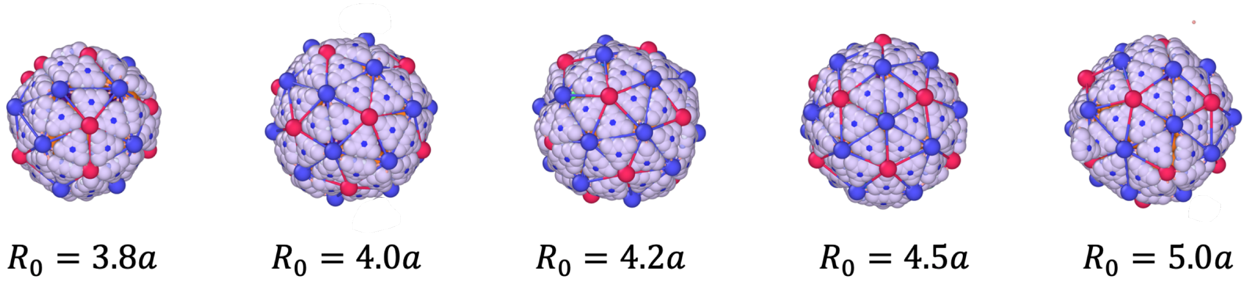

Figure S5: **Final assembly products at varying spontaneous curvatures.** The final assembly products of simulations using branchB polymer at the protein concentration $C_p = 200\mu M$ for various spontaneous radius of curvature $R_0 = 3.8a$, $4.0a$, $4.2a$, $4.5a$, and $5.0a$. Except for $R_0 = 3.8a$, all the other structures have icosahedral symmetry regardless of the preferred radius of curvature. The polymer length is $100a$, the protein-protein interaction strength is $\epsilon_{pp} = 5k_BT$ and the probability of transition from the elastic to rigid state is $p_R = 0.1$.

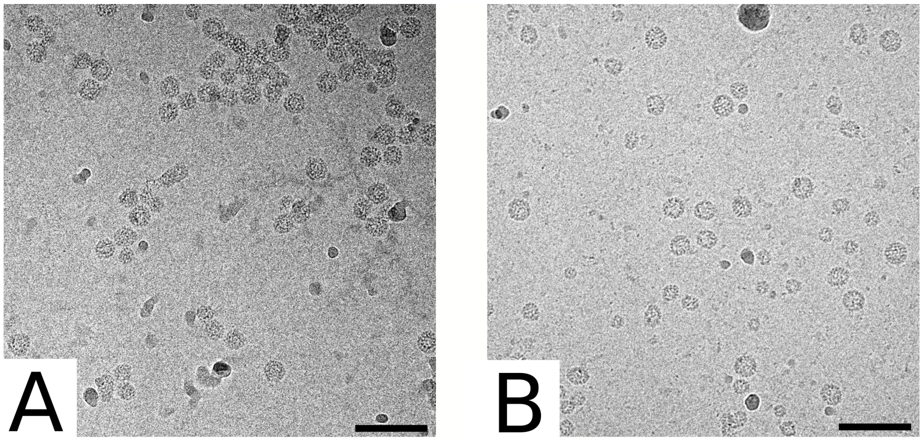

Figure S6: **CryoTEM images of structures obtained with RF2 (A) and C2 (B).** The concentration of CCMV capsid proteins is 75 μM and the protein-to-RNA mass ratio is 6.0. Structures with RF2 are slightly more aggregated than those with C2 and many of them are doublets or even multiplets. Note also that empty nanotubes are also encountered in both cases, possibly due to the excess of capsid proteins. Scale bar is 100 nm.

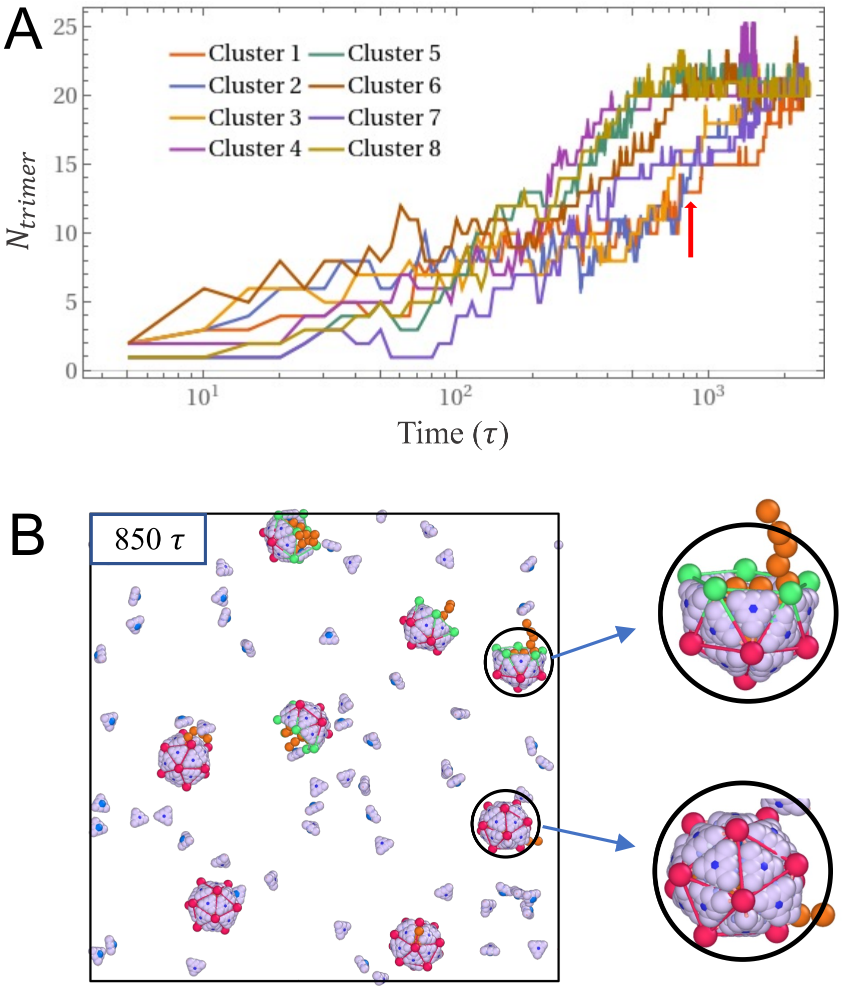

Figure S7: **Formation of $T = 1$ structures.** Assembly of several $T = 1$ structures in a mixture of short chains and trimers with a concentration of $C_p = 100\mu M$. (A) The number of trimers aggregated around each genome over time. (B) Simulation snapshot at $t = 850\tau$, where four particles form complete shells and four others form incomplete shells. The plots reveal that the simultaneous assembly of multiple $T = 1$ particles closely resembles that of a single one. It's worth noting that tracking the assembly pathways of $T = 3$ structures is challenging due to the prevalence of metastable structures. Consequently, we focused on monitoring the assembly of several $T = 1$ viruses. An important consideration is the stoichiometric ratio of protein subunits per genome, which dictates the formation of incomplete or overgrown shells.

Caption for Movie S1. **Assembly pathway of a $T = 3$ capsid. Protein trimers diffuse around a linear genome of length $80a$ and self-organize from an initially disordered complex into a complete icosahedral shell. The conformational switch (allosteric) probabilities between rigid (free) and elastic (bound) trimers are $p_E = 1.0$ and $p_R = 0.1$. Protein concentration is $C_p = 100\mu$M, the spontaneous radius of curvature of elastic trimers is $R_0 = 4.2a$, and the protein–protein interaction strength is $\epsilon_{pp} = 5.0k_BT$. The transition from disorder to order occurs over an extended timescale, as many subunits must dissociate and re-associate—even after the shell appears nearly complete—for the capsid to reach its final symmetric form. These transitions involve repeated switching between free subunits and those connected to the capsid through elastic bonds formed via allosteric conformational changes.**

Caption for Movie S2. **Assembly pathway of a $T = 3$ capsid under faster subunit detachment conditions. Protein trimers diffuse around a linear genome of length $80a$ and self-organize into a complete icosahedral shell. Compared to Movie 1, the detachment rate of subunits from the growing shell is higher due to an increased probability of allosteric switching from the elastic (bound) to the rigid (free) state ($p_R = 0.2$). To compensate for this increased detachment, the hydrophobic protein–protein interaction strength is raised to $\epsilon_{pp} = 6.0k_BT$. Together, these changes accelerate the assembly process and increase its efficiency. The probability of transitioning from rigid to elastic remains $p_E = 1.0$, and the spontaneous radius of curvature of elastic trimers is $R_0 = 4.2a$. Protein concentration is $C_p = 100\mu$M. As in Movie 1, the capsid assembles through repeated association and dissociation events, mediated by allosteric conformational changes and the formation of elastic bonds.**

Caption for Movie S3. **Assembly pathway of a $T = 4$ capsid. Protein trimers diffuse around a linear genome of length $80a$ and self-organize into a complete $T = 4$ icosahedral shell. The conformational switch (allosteric) probabilities between rigid (free) and elastic (bound) trimers are $p_E = 1.0$ and $p_R = 0.1$. Protein concentration is $C_p = 100\mu$M, the spontaneous radius of curvature of elastic trimers is $R_0 = 4.2a$, and the protein–protein interaction strength is $\epsilon_{pp} = 7.0k_BT$. Assembling a $T = 4$ shell is more challenging due to its larger size, and the system requires a longer timescale to transition from a disordered complex to a fully**

symmetric structure. The capsid forms through a dynamic process of subunit association and dissociation, mediated by allosteric conformational switching and elastic bond formation.

Caption for Movie S4. **Simultaneous formation of multiple $T = 1$ capsids. Protein trimers assemble around eight linear genomes, each of length $20a$, forming eight complete $T = 1$ icosahedral shells. The conformational switch (allosteric) probabilities between rigid (free) and elastic (bound) trimers are $p_E = 1.0$ and $p_R = 0.1$. Protein concentration is $C_p = 100\mu$M, the spontaneous radius of curvature of elastic trimers is $R_0 = 3.0a$, and the protein–protein interaction strength is $\epsilon_{pp} = 5.0k_BT$. The smaller genome length and higher curvature facilitate the rapid and parallel assembly of multiple capsids through dynamic association and allosterically driven conformational switching.**
